# Host-Parasite Incongruences in Rodent *Eimeria* Suggest Significant Role of Adaptation Rather than Cophylogeny in Maintenance of Host Specificity

**DOI:** 10.1371/journal.pone.0063601

**Published:** 2013-07-04

**Authors:** Jana Kvičerová, Václav Hypša

**Affiliations:** 1 Department of Parasitology, Faculty of Science, University of South Bohemia, Branišovská, České Budějovice, Czech Republic; 2 Biology Centre, Institute of Parasitology, Academy of Sciences of the Czech Republic, Branišovská, České Budějovice, Czech Republic; University of Heidelberg Medical School, Germany

## Abstract

The degree of host specificity, its phylogenetic conservativeness and origin are virtually unknown in *Eimeria*. This situation is largely due to the inadequate sample of eimerian molecular data available for reliable phylogenetic analyses. In this study, we extend the data set by adding 71 new sequences of coccidia infecting 16 small-mammal genera, mostly rodents. According to the respective feasibility of PCR gene amplification, the new samples are represented by one or more of the following genes: nuclear 18S rRNA, plastid ORF 470, and mitochondrial COI. Phylogenetic analyses of these sequences confirm the previous hypothesis that *Eimeria*, in its current morphology-based delimitation, is not a monophyletic group. Several samples of coccidia corresponding morphologically to other genera are scattered among the *Eimeria* lineages. More importantly, the distribution of eimerians from different hosts indicates that the clustering of eimerian species is influenced by their host specificity, but does not arise from a cophylogenetic/cospeciation process; while several clusters are specific to a particular host group, inner topologies within these clusters do not reflect host phylogeny. This observation suggests that the host specificity of *Eimeria* is caused by adaptive rather than cophylogenetic processes.

## Introduction

Specificity to a more or less restricted group of hosts is one of the fundamental characteristics of most parasitic taxa. In parasitological research, this trait has traditionally been considered highly conserved from a phylogenetic point of view. This idea has led to the establishment of a broad spectrum of concepts and methods dealing with coevolution/cospeciation between the host and parasite [1]–[6].

More recently, analyses based on molecular data have revealed a tendency toward the conservativeness of host specificity and even a strong cospeciation signal in many parasitic groups [2], [7], [8]. However, other studies have demonstrated that such conservativeness of host specificity is not the rule, and have found many surprising inconsistencies among host and parasite phylogenies [9]–[13]. Moreover, many other features presumed to be reliable determinants of taxonomy and classification, whether morphological or ecological, have been shown to suffer the same phylogenetic inconsistencies [14]–[19]. Consequently, the traditional classification of many taxa is artificial, many generic names do not designate monophyletic groups, and the significance of host specificity in parasite evolution remains unclear.

There is currently no consensus or general view as to the degree to which host specificity is phylogenetically conserved in various parasites. Apart from the many methodological problems presented by analyses of this feature [2], [20], one drawback is the traditional focus on just a few model groups, such as chewing lice, lice, and nematodes [7], [21]–[25], and a paucity of data to address host specificity in many others. The situation may be particularly difficult and the analyses misleading in species-rich taxa for which only poor sampling is currently available; any pattern observed within a phylogenetic background may only be the random outcome of inadequate arbitrary sampling rather than a reflection of real tendencies within a given group.

Considering their importance, it is quite surprising that coccidia of the genus *Eimeria* belong to an example of just such an inadequately analysed group. A majority of the traditional taxonomical studies on coccidia are based solely on the morphology of sporulated oocysts (e.g. [26]–[33]). Several others deal with host specificity (inferred mostly from laboratory cross-transmission studies) and pathogenicity of coccidia [34]–[37].

Few comprehensive molecular studies have been performed so far [38]–[41]. They have, however, shown that some morphological features of the oocyst (e.g. oocyst size, sporocyst size and length/width ratio) are phylogenetically inconsistent and cannot be used as taxonomic determinants. Several morphological studies have also indicated that these features even vary during the development/patency of the oocyst [42]–[44]. Moreover, the determination of “oocyst shape” is a subjective criterion that depends on the microscopic experience of the individual observer (e.g. oval vs. ovoidal vs. ellipsoidal shape; the “spherical” or “subspherical” shape is often determined in dependence on the angle of view). These factors are the main reasons for the unsatisfactory state of current eimerian taxonomy and evolutionary research. This problem is not restricted to phylogenetic relationships within *Eimeria*, but the whole genus has shown to be non-monophyletic; several species corresponding morphologically to other genera (e.g. *Caryospora*, *Cyclospora* and *Isospora*) branch within the *Eimeria* cluster. Similarly, *Isospora* is also clearly a polyphyletic genus, with several lineages scattered among Eimeriidae and some species belonging to Sarcocystidae [45]–[49].

The inadequacy of the available sampling for phylogenetic analyses has also hampered the evaluation of the significance of host specificity in eimerian evolution. Most of the genetic lineages designated as host-specific are derived from only a few closely related hosts. The only exceptions being the rodent-derived *Eimeria*, currently represented by a reasonable number of samples. The results obtained with these taxa indicate that most of the rodent eimerians fall into two unrelated host-specific lineages [50]–[52]. Most recently, *Eimeria myoxi* was found to be an exception, clustering outside these two rodent groups [53].

In this study, we further explore the phylogenetic significance of host specificity within *Eimeria* by adding 71 new coccidian sequences. Since the most frequently utilized phylogenetic marker, 18S rDNA, has proven to be unsufficient for this group, we also sequenced two additional DNA regions whenever possible: cytochrome c oxidase subunit I (COI) and ORF 470. To obtain a consistent picture, allowing for evolutionary inference, we mainly focused on the rodent-derived *Eimeria*; the complete set thus contains 44 eimerian parasites from various rodent groups from 8 families. This representative set demonstrates that with an increased number of available taxa, phylogenetic relationships become less host-dependent.

## Materials and Methods

### Sample Collection and Treatment

Rodents were trapped using classic wooden traps. This study was carried out in strict accordance with the current laws of the Czech Republic; animals were trapped under official permits from the Office for the South Bohemian Region, Department of the Environment, Agriculture and Forestry (Permit Number: KUJCK 11134/2010 OZZL/2/Ou) and the Ministry of the Environment of the Czech Republic (Permit Number: 27873/ENV/11). The protocol was approved by the Committee on the Ethics of Animal Experiments of the University of South Bohemia (Permit Number: 13841-11). Sampled animals do not represent protected species and private/protected land was not accessed during the field studies. Shrew, mole, mole-rat, and pangolin samples were obtained from already deceased animals.

The fresh faeces or gut content of each individual animal were placed into 4% (w/v) potassium dichromate solution (K_2_Cr_2_O_7_) and stored at 4°C. Faecal samples were examined for the presence of coccidian oocysts by the standard flotation technique with Sheather’s sucrose solution (sp.gr. 1.30). An Olympus BX51 microscope equipped with an Olympus Camedia C-5060W camera and Quick Photo Pro v. 2.0 PC software was used for species-specific identification of found oocysts. Morphological and morphometrical features were evaluated according to [54].

Coccidian genomic DNA was extracted using the FastDNA SPIN Kit for Soil (MP Biomedicals) according to the manufacturer’s instructions. Three different genes (nuclear 18S rRNA, plastid ORF 470 and mitochondrial COI) were amplified using the HotStarTaq DNA polymerase (Qiagen) and PCR protocols according to [41], [51] and [55]. PCR products of expected sizes (18S rDNA ∼1500 bp, ORF 470 ∼700 bp and COI ∼700 bp) were cloned into the pGEM-T Easy Vector (Promega). Five plasmid clones of each sample were obtained using the PureLink Quick Plasmid Miniprep Kit (Invitrogen). Plasmids were sequenced on an automatic 3730XL DNA analyser maintained by the Macrogen, Inc. (Korea) using PCR primers or specifically-designed internal primers [41], [51], [55]. Sequences were identified by BLAST analysis, edited using the DNASTAR program package (DNASTAR Inc.), and deposited to the NCBI GenBank database under the Accession numbers JQ993644-JQ993714.

### Phylogenetic Analyses

To explore phylogenetic signal from the obtained sequences in a complex way, we built several different single- and multi-gene matrices. Three single-gene matrices, *18S rDNA*, *COI*, and *ORF 470*, were created using different taxa samplings according to the availability of given sequences for individual taxa (Table 1). The *Skeleton* matrix included taxa for which all three genes were available. The *Concatenated* matrix encompassed all taxa for which at least one gene was available. To achieve stable and reliable placement of the root, multiple taxa were used as outgroups (Table 1). All matrices were aligned and analysed at the nucleotide level. Alignments were constructed in the MAFFT v. 6 program [56], [57] and corrected manually using the BioEdit program [58]. Maximum likelihood (ML) and Bayesian inference (BI) were used for phylogenetic analyses. The most suitable models of sequence evolution were identified with the jModelTest [59], [60] and MrModel [61] programs using Akaik’s criterion. ML was performed in Phyml v. 2.4.3 [62] with the GTR+Г+I model and parameters estimated from the data. BI was done using MrBayes v. 3.1.2 [63] with a GTR+Г+I model for 50 million generations. Chain convergence and burn-in were estimated according to the indices implemented in the MrBayes program (deviation of split frequencies, potential scale reduction factor – PSRF) and using the Tracer program [64]. The trees were summarized after removing 20% burn-in, visualized using TreeView v. 1.6.6 [65], and adjusted in Adobe Illustrator CS5 v. 15.0 (Adobe Systems Inc.). Phylogenetic data are accessible in the TreeBASE database, Study ID 12861.

**Table 1:** Taxa and sequences included in the phylogenetic analyses.

Organism	Acc. number *18S rDNA*	Acc. number *ORF 470*	Acc. number *COI*
*Eimeria acervulina*	U67115	–	FJ236419
*E. adenoeides*	AF324212	–	–
*E. ahsata*	AF338350	–	–
*E. alabamensis*	AF291427	–	–
*E. albigulae*	AF307880	AF311630	–
*E. antrozoi*	AF307876	–	–
*E. arizonensis*	AF307878	AF311631	–
*E. arnyi*	AY613853	–	–
*E. attwateri*	EU481858	–	–
*E. auburnensis*	AY876927	–	–
*E. auritusi*	DQ398107	–	–
***E. banffensis***	**JQ993644**	–	–
*E. bovis*	U77084	–	–
*E. brunetti*	U67116	–	–
***E. burdai*** *	**JQ993666**	**JQ993682**	**JQ993709**
***E. cahirinensis*** ** NFS**	**JQ993645**	–	**JQ993686**
***E. cahirinensis*** ** SFS**	**JQ993646**	–	–
***E. cahirinensis*** ** WR**	**JQ993647**	–	**JQ993687**
***E. callospermophili***	**JQ993648**	–	**JQ993688**
*E. catronensis*	AF324213	–	–
***E. caviae*** *	**JQ993649**	**JQ993672**	**JQ993689**
*E.* cf. *mivati*	FJ236378	–	FJ236441
*E. chaetodipi*	AF339489	–	–
***E. chinchillae***	**JQ993650**	–	–
*E. chobotari*	AF324214	–	–
***E. coecicola***	EF694015	–	**JQ993690**
*E. crandallis*	AF336339	–	–
*E. cylindrica*	AY876928	–	–
*E. dipodomysis*	AF339490	–	–
*E. ellipsoidalis*	AY876929	–	–
***E. exigua*** *	EF694007	**JQ993673**	**JQ993691**
*E. falciformis*	AF080614	AF311632	–
*E. faurei*	AF345998	–	–
***E. flavescens*** *	EF694011	JF304149	**JQ993692**
*E. furonis*	AB239130	–	–
*E. gruis*	AB205165	–	–
***E. intestinalis*** *	EF694012	**JQ993674**	**JQ993693**
***E. irresidua*** *	EF694009	**JQ993675**	**JQ993694**
*E. langebarteli*	AF311640	AF311639	–
*E. leucopi*	AF339491	–	–
***E. magna*** *	EF694016	JF304150	**JQ993695**
*E. maxima*	DQ538348	–	FJ236459
***E. media***	EF694013	**JQ993676**	–
*E. meleagrimitis*	AF041437	–	–
*E. mitis*	U40262	–	–
*E. mivati*	U76748	–	EF174185
***E. myoxi*** *	JF304148	JF304151	**JQ993696**
***E. nafuko***	**JQ993665**	–	**JQ993708**
*E. necatrix*	DQ136185	–	EU025108
*E. nieschulzi*	U40263	AF311633	–
***E.*** ** sp. ex ** ***Phataginus tricuspis*** *	**JQ993651**	**JQ993677**	**JQ993697**
*E. onychomysis*	AF307879	AF311634	–
*E. ovinoidalis*	AF345997	–	–
*E. papillata*	AF311641	AF311635	–
*E. perforans*	EF694017	–	–
*E. peromysci*	AF339492	–	–
*E. phalacrocoraxae*	DQ398106	–	–
*E. pilarensis*	AF324215	–	–
***E. piriformis***	EF694014	–	**JQ993698**
*E. polita*	AF279667	–	–
*E. porci*	AF279666	–	–
*E. praecox*	U67120	–	–
*E. ranae*	EU717219	–	–
*E. reedi*	AF311642	AF311636	–
*E. reichenowi*	AB205175	–	–
*E. rioarribaensis*	AF307877	–	–
*E. scabra*	AF279668	–	–
*E. scholtysecki*	AF324216	–	–
*E. separata*	AF311643	AF311637	–
*E. sevilletensis*	AF311644	AF311638	–
***E. stiedai***	EF694008	**JQ993678**	–
*E. subspherica*	AY876930	–	–
***E. synaptomysis***	**JQ993652**	–	–
*E. telekii*	AF246717	–	–
*E. tenella* *	U67121	Y12333	FJ236458
*E. trichosuri*	FJ829323	–	–
*E. tropidura*	AF324217	–	–
***E. vejdovskyi***	EF694010	–	**JQ993699**
***E. vilasi***	**JQ993653**	–	–
*E. weybridgensis*	AY028972	–	–
*E. wyomingensis*	AY876931	–	–
*E. zuernii*	AY876932	–	–
*E.* sp. DAM-2009	FN298443	–	–
*E.* sp. ESP-181	AB447983	–	–
*E.* sp. TKC-1-2005	DQ072716	–	–
*E.* sp. TKC-2-2005	DQ167480	–	–
***E.*** ** sp. ex ** ***Acomys*** ** sp. K2**	**JQ993654**	–	–
***E.*** ** sp. ex ** ***A. agrarius*** ** 21439**	**JQ993655**	–	–
***E.*** ** sp. ex ** ***A. agrarius*** ** 21455**	**JQ993656**	–	–
***E.*** ** sp. ex ** ***A. agrarius*** ** 21615**	**JQ993657**	–	–
***E.*** ** sp. ex ** ***A. agrarius*** ** 21617** *	**JQ993658**	**JQ993679**	**JQ993700**
***E.*** ** sp. ex ** ***A. agrarius*** ** 21655** *	**JQ993659**	**JQ993680**	**JQ993701**
***E.*** ** sp. ex ** ***A. agrarius*** ** 21668**	**JQ993660**	–	**JQ993702**
***E*** **. sp. ex ** ***A. flavicollis*** ** 1**	–	–	**JQ993703**
***E*** **. sp. ex ** ***A. flavicollis*** ** 4**	–	–	**JQ993704**
***E*** **. sp. ex ** ***A. flavicollis*** ** 12**	–	–	**JQ993705**
***E.*** ** sp. ex ** ***A. sylvaticus*** ** 08/50**	**JQ993661**	–	**JQ993706**
***E.*** ** sp. ex ** ***A. sylvaticus*** ** 08/53** *	**JQ993662**	**JQ993681**	**JQ993707**
***E.*** ** sp. ex ** ***C. cricetus*** ** K7**	**JQ993663**	–	–
***E.*** ** sp. ex ** ***G. dasyurus***	**JQ993664**	–	–
***E.*** ** sp. ex ** ***M. natalensis***	**JQ993667**	–	–
***E.*** ** sp. ex ** ***S. araneus*** ** 136**	–	**JQ993683**	**JQ993710**
*Caryospora bigenetica*	AF060975	–	–
*Choleoeimeria* sp.	AY043207	–	–
*Cyclospora cayetanensis*	AF111183	–	–
*C. cercopitheci*	AF111184	–	–
*C. colobi*	AF111186	–	–
*C. papionis*	AF111187	–	–
*Cystoisospora belli* •	AF106935	–	–
*C. felis* •	L76471	–	–
*C. ohioensis* •	AF029303	–	–
*C. orlovi* •	AY365026	–	–
*C. rivolta* •	AY618554	–	–
*C. suis* •	U97523	–	–
*C. timoni* •	AY279205	–	–
*Goussia janae*	AY043206	–	–
*G. metchnikovi*	FJ009244	–	–
*G. neglecta*	FJ009242	–	–
*G. noelleri*	FJ009241	–	–
*G.* ex *Bufo bufo*	FJ009243	–	–
Intranuclear coccidium JW-2004	AY728896	–	–
**coccidium ex ** ***C. cricetus*** ** K4**	**JQ993668**	**JQ993684**	–
*Isospora gryphoni*	AF080613	–	–
*I. robini*	AF080612	–	–
*Isospora* sp. iSAT1	–	–	FJ269357
*Isospora* sp. iSAT2	–	–	FJ269358
*Isospora* sp. iSAT3	–	–	FJ269359
*Isospora* sp. iSAT4	–	–	FJ269360
*Isospora* sp. iSAT5	–	–	FJ269361
*Isospora* sp. iSAT6	–	–	FJ269362
***I.*** ** sp. ex ** ***A. flavicollis*** ** B13**	–	–	**JQ993711**
***I.*** ** sp. ex ** ***Talpa*** ** 106**	**JQ993669**	–	**JQ993712**
***I.*** ** sp. ex ** ***Talpa*** ** 151**	**JQ993670**	–	**JQ993713**
***I.*** ** sp. ex ** ***Talpa*** ** 158**	**JQ993671**	–	–
***I.*** ** sp. ex ** ***Talpa*** ** 218**	–	**JQ993685**	**JQ993714**
*Toxoplasma gondii* •	M97703	U87145	DQ228959

* : sequences included in the *Skeleton* matrix.

•: taxa used as outgroups for the phylogenetic analyses.

– : the sequence is not available.

Taxa for which new sequences were obtained in this study and Accession numbers of these sequences are printed in bold.

## Results

**Figure 1:**
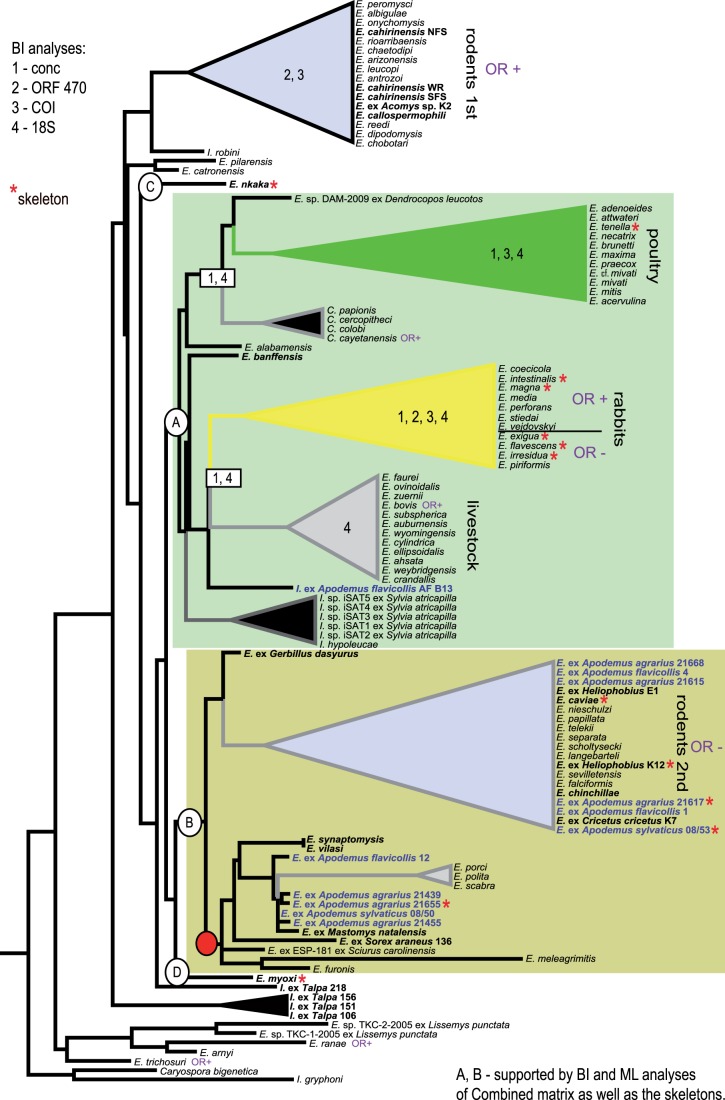
*Concatenated* ML tree. Letters A–D indicate clusters delimited according to the *Skeleton* tree (taxa present in the *Skeleton* tree are labeled with asterisks). Clades A and B are supported by both BI and ML analyses of the *Concatenated* and *Skeleton* matrices. The red node indicates a cluster with weak host specificity. Numbers 1–4 indicate lineages that are also supported by BI analyses of the following matrices: 1, *Concatenated*; 2, *ORF 470*; 3, *COI*; 4, *18S rDNA*. The newly added samples are printed in bold; coccidia from rodents are printed in blue. To decrease the size of the tree for the printed presentation, we removed several of the most basal outgroups.

**Figure 2:**
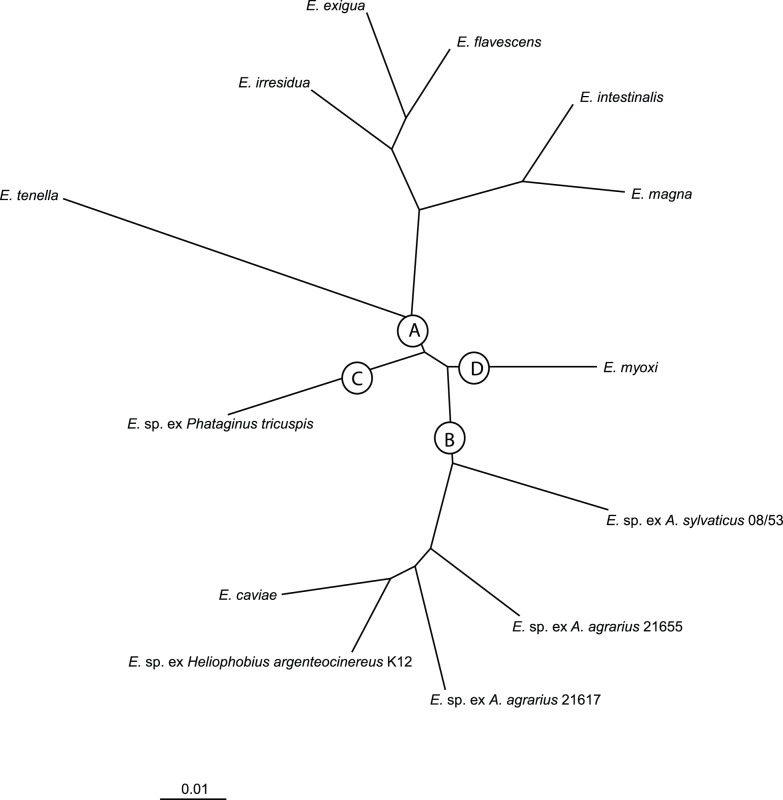
A *Skeleton* tree. *Skeleton* tree (ML and BI) of the taxa for which all 3 genes (*18S rDNA*, *ORF 470* and *COI*) are available.

While the trees obtained via phylogenetic analyses with different data sets and methods vary in the positions of individual branches, they are compatible in their overall structure and arrangement (Figs. 1, S1, S2, S3, S4, S5, S6, S7, S8). Since the aim of this study was to analyse the monophyly and composition of whole clusters characterized by various biological features (e.g. morphology, host specificity, geographic origin) rather than relationships among individual species, we focused on the comparison of particular internal nodes in the obtained trees. To allow for a transparent comparison among the trees constructed from different data sets, we established a specific reference method. We chose the *Concatenated* ML tree (Fig. 1) to delimit two types of clusters. First, we labeled all monophyletic groups that were characterized by a well-defined spectrum of host taxa (vertical lines in the Fig. 1); second, we “fixed” all nodes that were strongly supported by the bootstrap values and were also preserved in the BI tree (open squares at the branches; Fig. 1). We then identified whether each of these “fixed” groups is represented by at least one sample in the *Skeleton* tree (asterisks next to taxa names in Fig. 1). The *Skeleton* tree divides the included taxa into 4 main arbitrarily-delimited clades (A–D; Fig. 2). When fixed according to the *Skeleton* taxa, these clades are also preserved and well-supported in all performed single-gene analyses and in the *Concatenated* trees (Figs. 1, S1, S2, S3, S4, S5, S6, S7, S8).

The single-gene trees as well as the *Concatenated* trees also demonstrate that whereas some genera (e.g. *Cyclospora*) are monophyletic, others (*Eimeria* and *Isospora*) are polyphyletic (Figs. 1, S1, S2, S3, S4, S5, S6). In all analyses performed, the rodent *Eimeria* species are divided into several (6–8) paraphyletic lineages. The composition of these clades corresponds to the presence/absence of the oocyst residuum (OR) (Fig. 1). Other criteria (oocyst shape and size, presence/absence of a micropyle and other inner oocyst structures, location of endogenous development, pre-patent and patent periods, sporulation time), if known for the studied taxa, do not correlate with the topology (Table 2). Of our new rodent samples, three species from the newly added hosts fall within the OR+ rodent cluster (namely *E. cahirinensis*, *E. callospermophili* and *Eimeria* sp. from *Acomys* sp.). Another twelve samples (e.g. *E. caviae*, *E. chinchillae*, *Eimeria* spp. from *Apodemus* spp., *Cricetus cricetus*, *Heliophobius argenteocinereus*, *Mastomys natalensis*) branched within the OR- rodent cluster (Fig. 1). While most of *Eimeria* tend to cluster according to the host (e.g. distinct and stable fowl-, wild living bird-, porcine-, bovine-, rabbit- and rodent- lineages), the *Concatenated* tree also indicates that the sampling is still insufficient and several taxa lack a clear phylogenetic position (e.g. eimerians from the tree pangolin, garden dormouse, sheep, ferret and marsupials) (Fig. 1).

**Table 2:** Morphological features and origin of the newly obtained samples within this study.

Species of *Eimeria*	Oocyst shape	Oocyst size	OW	OR	MP	Host species	Host taxonomy	Origin
*E.* sp. ex *Acomys* sp. K2	ellipsoidal	16–27×15–22	slightly pitted	**+**	−	*Acomys* sp.	Rodentia: Muridae	Kenya, Eastern Province
*E.* sp. ex *Apodemus agrarius* 21439	ovoid-ellipsoidal	20×18	rough	–	–	*Apodemus agrarius*	Rodentia: Muridae	SK, Rozhanovce
*E.* sp. ex *Apodemus agrarius* 21455	ovoid-ellipsoidal	21–24×18–20	rough	–	–	*Apodemus agrarius*	Rodentia: Muridae	SK, Rozhanovce
*E.* sp. ex *Apodemus agrarius* 21615	ovoid	17–22×15–18	smooth	–	–	*Apodemus agrarius*	Rodentia: Muridae	SK, Šebastovce
*E.* sp. ex *Apodemus agrarius* 21617	ovoid	17–19×13–17	smooth	–	–	*Apodemus agrarius*	Rodentia: Muridae	SK, Šebastovce
*E.* sp. ex *Apodemus agrarius* 21655	ellipsoidal	25–30×18–20	smooth	–	**+**	*Apodemus agrarius*	Rodentia: Muridae	SK, Rozhanovce
*E.* sp. ex *Apodemus agrarius* 21668	ellipsoidal	24–28×16–18	smooth	–	**+**	*Apodemus agrarius*	Rodentia: Muridae	SK, Rozhanovce
*E.* sp. ex *Apodemus flavicollis* 1	ellipsoidal	20–24×13–17	smooth	–	–	*Apodemus flavicollis*	Rodentia: Muridae	CZ, Solany
*E.* sp. ex *Apodemus flavicollis* 4	ellipsoidal	24–28×17–20	smooth	–	–	*Apodemus flavicollis*	Rodentia: Muridae	CZ, Boršov nad Vltavou
*E.* sp. ex *Apodemus flavicollis* 12	broadly ellipsoidal	22–25×20–22	rough, pitted	**+**	–	*Apodemus flavicollis*	Rodentia: Muridae	CZ, Doupov
*E.* sp. ex *Apodemus sylvaticus* 08/50	ovoid- subspherical	19–23×16–19	rough	–	–	*Apodemus sylvaticus*	Rodentia: Muridae	UK, Ashford
*E.* sp. ex *Apodemus sylvaticus* 08/53	ellipsoidal	22–26×16–18	smooth	–	–	*Apodemus sylvaticus*	Rodentia: Muridae	UK, Ashford
*E. banffensis*	spherical-subspherical	27–32×24–28	rough	–	–	*Ochotona hyperborea*	Lagomorpha: Ochotonidae	Russia, Siberia
*E. cahirinensis* NFS	ellipsoidal-subspherical	20–30×19–25	pitted	+	–	*Acomys dimidiatus*	Rodentia: Muridae	Israel, Evolution Canyon, NFS
*E. cahirinensis* SFS	ellipsoidal-subspherical	19–28×17–23	slightly pitted	+	–	*Acomys dimidiatus*	Rodentia: Muridae	Israel, Evolution Canyon, SFS
*E. cahirinensis* WR	ellipsoidal-subspherical	22–29×18–24	slightly pitted	+	–	*Acomys dimidiatus*	Rodentia: Muridae	Jordan, Wadi Ramm
*E. callospermophili*	subspherical	15–19×15–18	smooth	+	–	*Spermophilus citellus*	Rodentia: Sciuridae	CZ, Chramosty-Líchovy
*E. caviae*	ovoid-ellipsoidal	19–25×17–20	smooth	–	–	*Cavia porcellus*	Rodentia: Caviidae	CZ, České Budějovice
*E. chinchillae*	ellipsoidal, flattened at poles	12–17×13–20	smooth	–	–	*Chinchilla laniger*	Rodentia: Muridae	CZ, České Budějovice
coccidium ex *Cricetus cricetus* K4	ovoid	10–11×8–10	smooth	–	–	*Cricetus cricetus*	Rodentia: Cricetidae	CZ, Velké Pavlovice
*E.* sp. ex *Cricetus cricetus* K7	ovoid-ellipsoidal	19–23×17–18	smooth	–	–	*Cricetus cricetus*	Rodentia: Cricetidae	CZ, Velké Pavlovice
*E. exigua*	spherical- subspherical	10–18×11–16	smooth	–	–	*Oryctolagus cuniculus*	Lagomorpha: Leporidae	CZ, České Budějovice
*E. flavescens*	ovoid	25–35×18–24	smooth	–	+	*Oryctolagus cuniculus*	Lagomorpha: Leporidae	CZ, České Budějovice
*E.* sp. ex *Gerbillus dasyurus*	ellipsoidal	26–30×20–24	rough	+	–	*Gerbillus dasyurus*	Rodentia: Gerbillidae	Jordan
*E. nafuko*	subspherical	15–16×12–13	smooth	–	–	*Heliophobius argenteocinereus*	Rodentia: Bathyergidae	CZ, České Budějovice
*E. burdai*	subspherical to broadly ellipsoidal	16–19×12–15	smooth	–	–	*Heliophobius argenteocinereus*	Rodentia: Bathyergidae	CZ, České Budějovice
*E. intestinalis*	piriform	22–30×16–21	smooth	+	+	*Oryctolagus cuniculus*	Lagomorpha: Leporidae	CZ, České Budějovice
*E. irresidua*	ovoid-barrel shaped	31–44×20–27	smooth	–	+	*Oryctolagus cuniculus*	Lagomorpha: Leporidae	CZ, České Budějovice
*E. magna*	ellipsoidal-ovoid	31–42×20–28	smooth	+	+	*Oryctolagus cuniculus*	Lagomorpha: Leporidae	CZ, České Budějovice
*E.* sp. ex *Mastomys natalensis*	ellipsoidal	18–30×12–20	granulated	+/−?	–	*Mastomys natalensis*(exp. *Mastomys coucha*)	Rodentia: Muridae	Malawi, Mulanje-Chitakali
*E. myoxi*	subspherical	16–20×15–18	slightly pitted	–	–	*Eliomys quercinus*	Rodentia: Gliridae	CZ, Šumava
*E.* sp. ex *Phataginus tricuspis*	spherical-broadly elliptical	14–22×13–18	rough	–	–	*Phataginus tricuspis*	Pholidota: Manidae	Angola, Cabinda Province
*E. synaptomysis*	ovoid-ellipsoidal	26–29×20–22	rough	–	–	*Lemmus trimucronatus*	Rodentia: Muridae	USA, Alaska
*E.* sp. ex *Sorex araneus* 136	spherical-subspherical	17–23×16–21	smooth	–	–	*Sorex araneus*	Insectivora: Soricidae	CZ, Boršov-Březí
*E. vilasi*	subspherical-ellipsoidal	12–23×7–19	smooth	–	–	*Spermophilus elegans*	Rodentia: Sciuridae	USA, Wyoming
*Isospora* sp. ex *Apodemus flavicollis* B13	spherical-subspherical	18,5×18,0	smooth	–	–	*Apodemus flavicollis*	Rodentia: Muridae	CZ, Litvínov
*I.* sp. ex *Talpa europaea* 106	ovoid-ellipsoidal-piriform	12–19×8–11	smooth, thin	–	–	*Talpa europaea*	Insectivora: Talpidae	CZ, Čejkovice (České Budějovice)
*I.* sp. ex *Talpa europaea* 151	ellipsoidal-piriform	13–20×8–12	smooth, thin	–	–	*Talpa europaea*	Insectivora: Talpidae	CZ, Hojná Voda
*I.* sp. ex *Talpa europaea* 158	ellipsoidal-piriform	12–17×8–11	smooth, thin	–	–	*Talpa europaea*	Insectivora: Talpidae	CZ, Klentnice (Pálava)
*I.* sp. ex *Talpa europaea* 218	oval-ellipsoidal	10–12×8–11	smooth, thin	–	–	*Talpa europaea*	Insectivora: Talpidae	CZ, Zálesí u Strakonic

CZ – Czech Republic, SK – Slovakia, UK – England; OW – oocyst wall, MP – micropyle, OR – oocyst residuum.

## Discussion

This study provides the most current insight into the phylogeny of eimerian parasites. Altogether 71 new sequences of coccidians obtained from 16 small-mammal genera (8 rodent-, 2 insectivore-, 2 lagomorph- and 1 manid- families) and 8 new *Isospora* sequences were analysed together with 124 coccidian sequences available from NCBI GenBank. Two main conclusions arise from the presented results. Firstly, they confirm the previous suggestion that *Eimeria*, in its current morphology-based delimitation, is not a monophyletic group. Secondly, and more importantly, they show an interesting relationship between host specificity and phylogeny: the distribution of eimerians from different hosts indicates that the clustering of eimerian species is influenced by their host specificity, but does not stem from a cophylogenetic process. Before attempting any serious evolutionary conclusion, however, it should be noted that the current sample of molecularly characterized *Eimeria* spp. and the spectrum of their available genes is extremely poor and inconsistent. Nevertheless, both of the main conclusions stated above are well-supported by all data and analyses.

The non-monophyletic nature of the genus *Eimeria* has been indicated by several previous studies [39], [40], [66]. It has brought forth the inconsistency between various phenotypic traits, most typically oocyst morphology, and phylogenetic relationships [14], [15], [41], [45]. However unnerving this finding may have been for the coccidian taxonomists, it is hardly surprising as a similar decoupling of the morphology of resistant stages and phylogenetic positions was also demonstrated in other parasites, for example Myxosporea [18].

This situation poses a serious problem for the future reclassification of the family Eimeriidae. Several species corresponding morphologically to different genera (e.g. *Caryospora*, *Cyclospora* and *Isospora*) branch within the *Eimeria* cluster. For example, *Isospora* is undoubtedly polyphyletic, with several lineages scattered among Eimeriidae and some among Sarcocystidae (Figs. S1, S2, S3, S4, [45]–[49]). However, sporulated oocysts of *Isospora* spp. are morphologically quite uniform (for examples, see [26] and/or [67]). Nevertheless, the genus *Isospora* has recently been divided into 2 separate genera according to their phylogeny, host specificity, and the presence/absence of a Stieda body (SB). Bird-associated *Isospora* (former *Atoxoplasma*) with SB belong to Eimeriidae and mammal-associated *Cystoisospora* lacking SB are members of Sarcocystidae [16], [45], [68]. However, it is important to point out that only 10 *Isospora*/*Cystoisospora* species from mammals (mainly cats and dogs) out of >130 described species [69] have been sequenced thus far. Moreover, comprehensive descriptions including photomicrographs show that several *Isospora* species infecting mammals, namely moles and shrews, evidently possess a conspicuous SB [67]. Sequences from these species could potentially bring new, unexpected insight into coccidian phylogeny. Regarding *Cyclospora*, only sequences of species infecting man, primates and dairy cattle are currently available, while the inclusion of additional *Cyclospora* species from other hosts (e.g. insectivores or reptiles) may bring more surprises.

Compared to the taxonomical questions, the issue of host specificity and its phylogenetic significance has been little explored in previously published studies. One of the main reasons for this deficiency is an inadequate representation of the host-specific groups. Only the group of rodent *Eimeria* is currently represented by a reasonable number and diversity of samples, whereas the other so-called host-specific lineages are mostly derived from very closely related hosts or even a single host species. Alternatively, they are defined by various artificial rather than taxonomic characteristics of their hosts (e.g. poultry parasites, livestock parasites, etc.).

Previous phylogenetic studies tended to group rodent-specific *Eimeria* species into two distant but monophyletic clusters with an unclear dependency on the taxonomic position of the hosts [50]–[52], [70]. Taking the number of eimerian samples from rodents and the taxonomic diversity of their hosts into account, these two clusters could be potentially envisaged as the two main evolutionary sources of rodent eimerians. The identification of a third lineage formed by *Eimeria myoxi* has suggested that the situation may be more complex [53]. The 26 new rodent-derived *Eimeria* samples added in this study further support this view. While many of the new samples from so far unexplored hosts (e.g. black-bellied hamster, chinchilla, ground squirrel, guinea pig, mole-rats, spiny mice, and several field mice) clearly belong to the two previously established rodent clades [50], [51], the position of others (garden dormouse, gerbil, multimammate rat, and some field mice) is more variable. It is also interesting to note that no rodent sample of *Eimeria*–like morphology falls into the A group (Fig. 1), containing mainly parasites from poultry, livestock, rabbits, and the isosporan lineage; the only *Apodemus*–isolated sample branching in this group clearly exhibits *Isospora* morphology (Fig. 1).

The relationship between host specificity and phylogeny displays an interesting pattern. While host specificity provides useful characteristics for many clusters (livestock, pigs, poultry, or rabbits), species arrangements within the clusters do not show any correlation with host phylogenies. The host conservativeness of the clusters is thus likely to reflect ecological, physiological, or other adaptations to a particular host group rather than host-parasite cospeciation.

Perhaps the most surprising outcome of this study is the phylogenetic diversity of *Eimeria* samples obtained from the genus *Apodemus*. While the exact taxonomic status of the 11 analysed samples and their precise position may not be entirely clear from the available topologies, they demonstrably cluster at least at four different places in the tree and cover quite a large phylogenetic span (Figs. 1, S1, S2). This result suggests that apart from the taxonomically representative sample of the hosts, knowledge of eimerian diversity from a single host genus or species represents yet another informative character. Considering the composition of the available data set, with only rodents sufficiently sampled in respect to taxonomic-representativeness as well as parasite diversity within a single host species, the trends revealed in this study should not be generalized. However, they do represent an intriguing research direction that needs to be addressed by obtaining representative samples from other host groups.

## Supporting Information

Figure S1 ***Concatenated*** **ML tree.** Strongly supported nodes (bootstrap supports >80%) are denoted by solid red circles. Nodes with bootstrap supports of 50–79% are marked with solid blue circles.
(PDF)

Figure S2 ***Concatenated*** **BI tree.** Strongly supported nodes (posterior probabilities >80%) are denoted by solid red circles. Nodes with posterior probabilities of 50–79% are marked with solid blue circles.
(PDF)

Figure S3 ***18S rDNA*** **ML tree.** Strongly supported nodes (bootstrap supports >80%) are denoted by solid red circles. Nodes with bootstrap supports of 50–79% are marked with solid blue circles.
(PDF)

Figure S4 ***18S rDNA*** **BI tree.** Strongly supported nodes (posterior probabilities >80%) are denoted by solid red circles. Nodes with posterior probabilities of 50–79% are marked with solid blue circles.
(PDF)

Figure S5 ***COI*** **ML tree.** Strongly supported nodes (bootstrap supports >80%) are denoted by solid red circles. Nodes with bootstrap supports of 50–79% are marked with solid blue circles.
(PDF)

Figure S6 ***COI*** **BI tree.** Strongly supported nodes (posterior probabilities >80%) are denoted by solid red circles. Nodes with posterior probabilities of 50–79% are marked with solid blue circles.
(PDF)

Figure S7 ***ORF 470*** **ML tree.** Strongly supported nodes (bootstrap supports >80%) are denoted by solid red circles. Nodes with bootstrap supports of 50–79% are marked with solid blue circles.
(PDF)

Figure S8 ***ORF 470*** **BI tree.** Strongly supported nodes (posterior probabilities >80%) are denoted by solid red circles. Nodes with posterior probabilities of 50–79% are marked with solid blue circles.
(PDF)
