# Supplementary figures and images for: Host-Parasite Incongruences in Rodent Eimeria Suggest Significant Role of Adaptation Rather than Cophylogeny in Maintenance of Host Specificity
Source: PLoS One. 2013 Jul 4;8(7):e63601. doi: 10.1371/journal.pone.0063601 (PMC3701668; doi:10.1371/journal.pone.0063601)

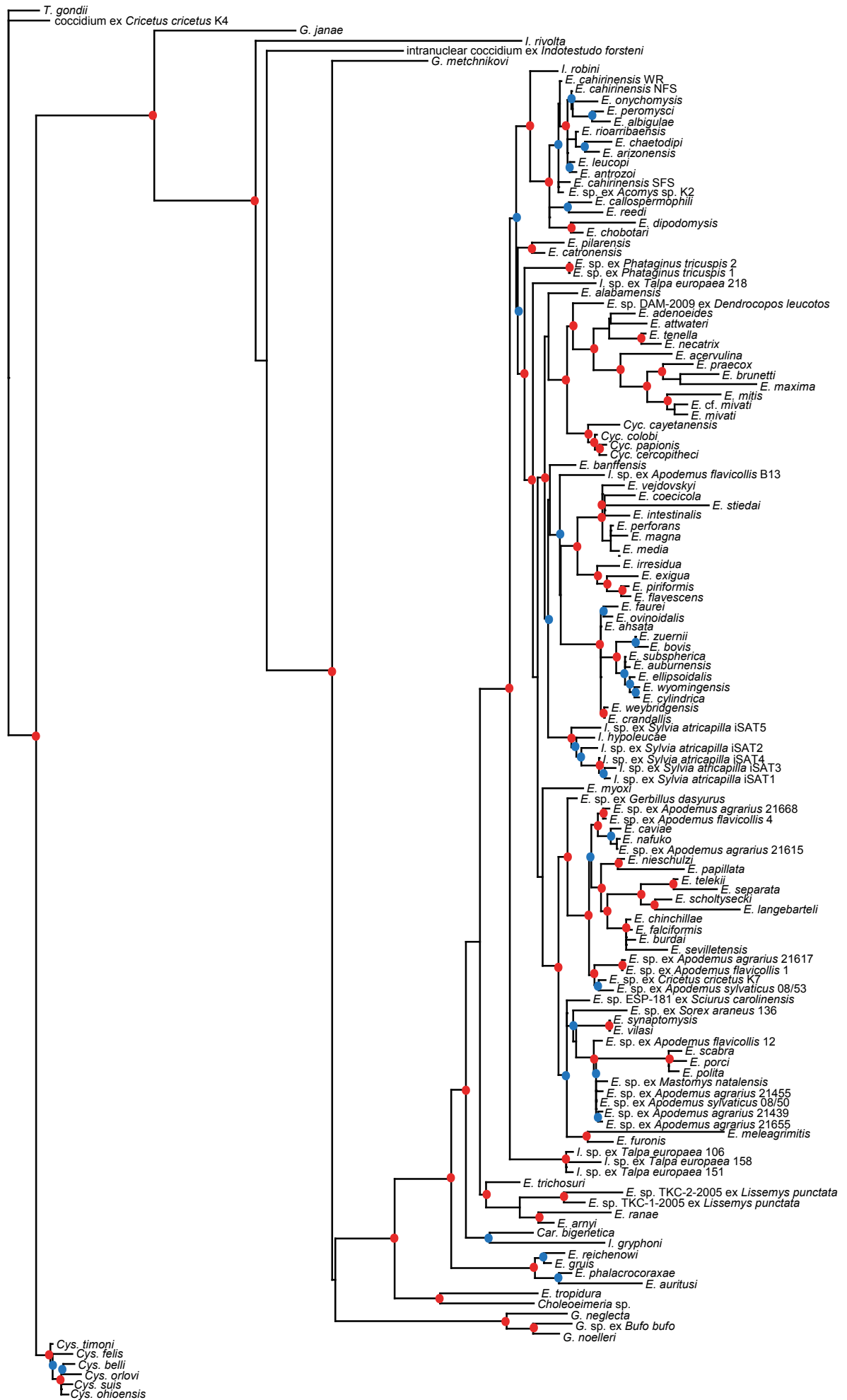

0.1

Supplement: Figure S1 — Concatenated ML tree. Strongly supported nodes (bootstrap supports >80%) are denoted by solid red circles. Nodes with bootstrap supports of 50–79% are marked with solid blue circles. (PDF) [file pone.0063601.s001.pdf]

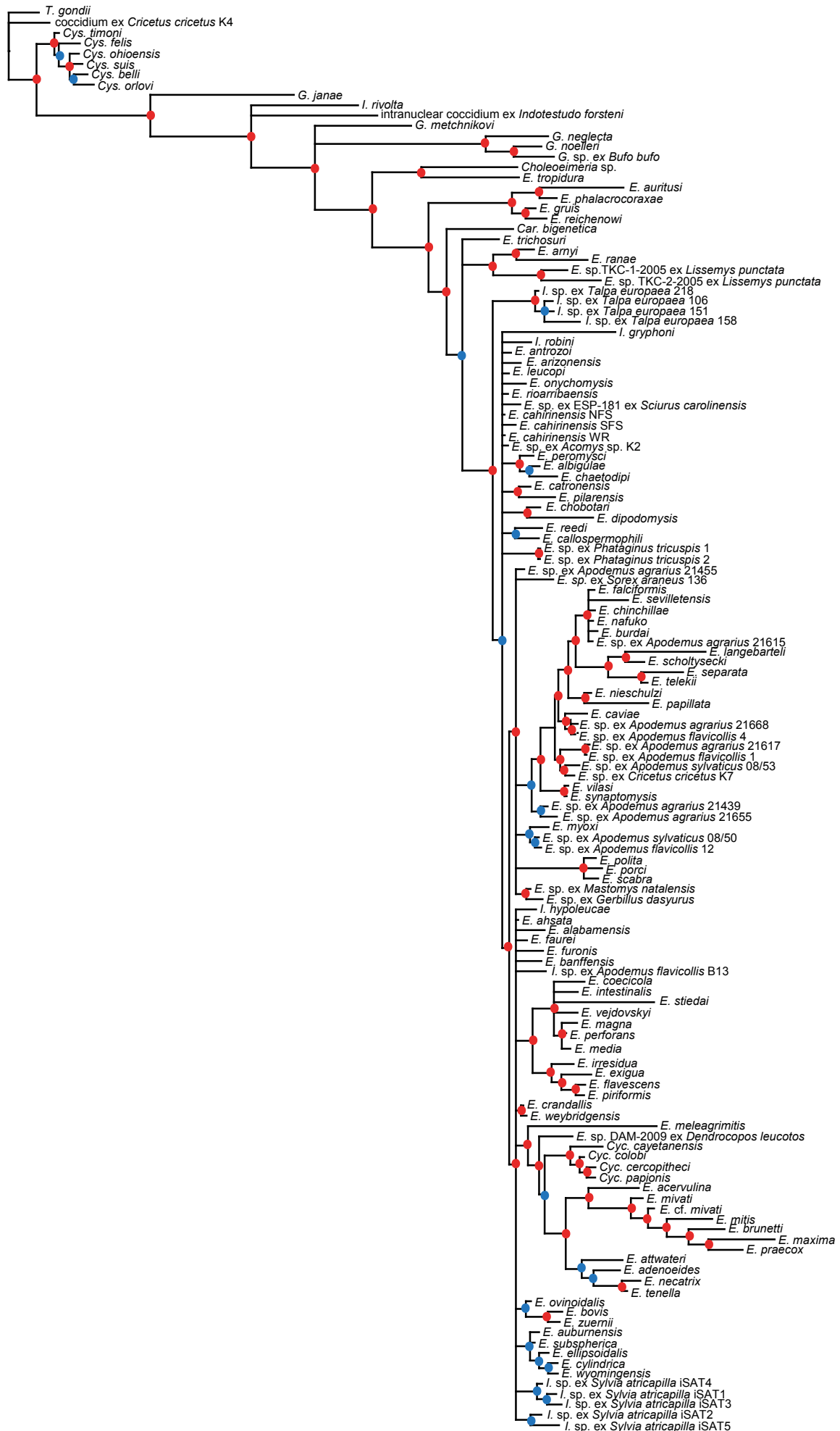

Supplement: Figure S2 — Concatenated BI tree. Strongly supported nodes (posterior probabilities >80%) are denoted by solid red circles. Nodes with posterior probabilities of 50–79% are marked with solid blue circles. (PDF) [file pone.0063601.s002.pdf]

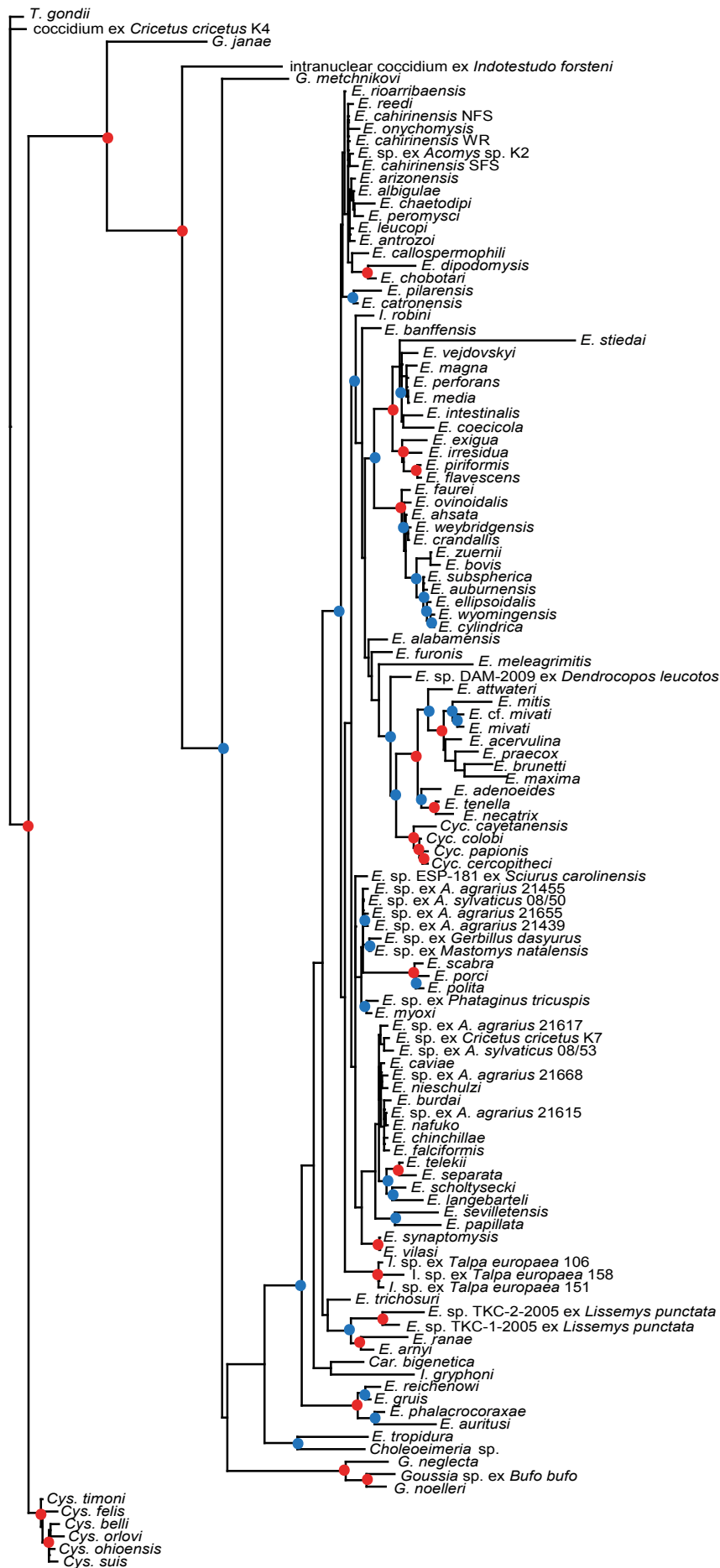

Supplement: Figure S3 — 18S rDNA ML tree. Strongly supported nodes (bootstrap supports >80%) are denoted by solid red circles. Nodes with bootstrap supports of 50–79% are marked with solid blue circles. (PDF) [file pone.0063601.s003.pdf]

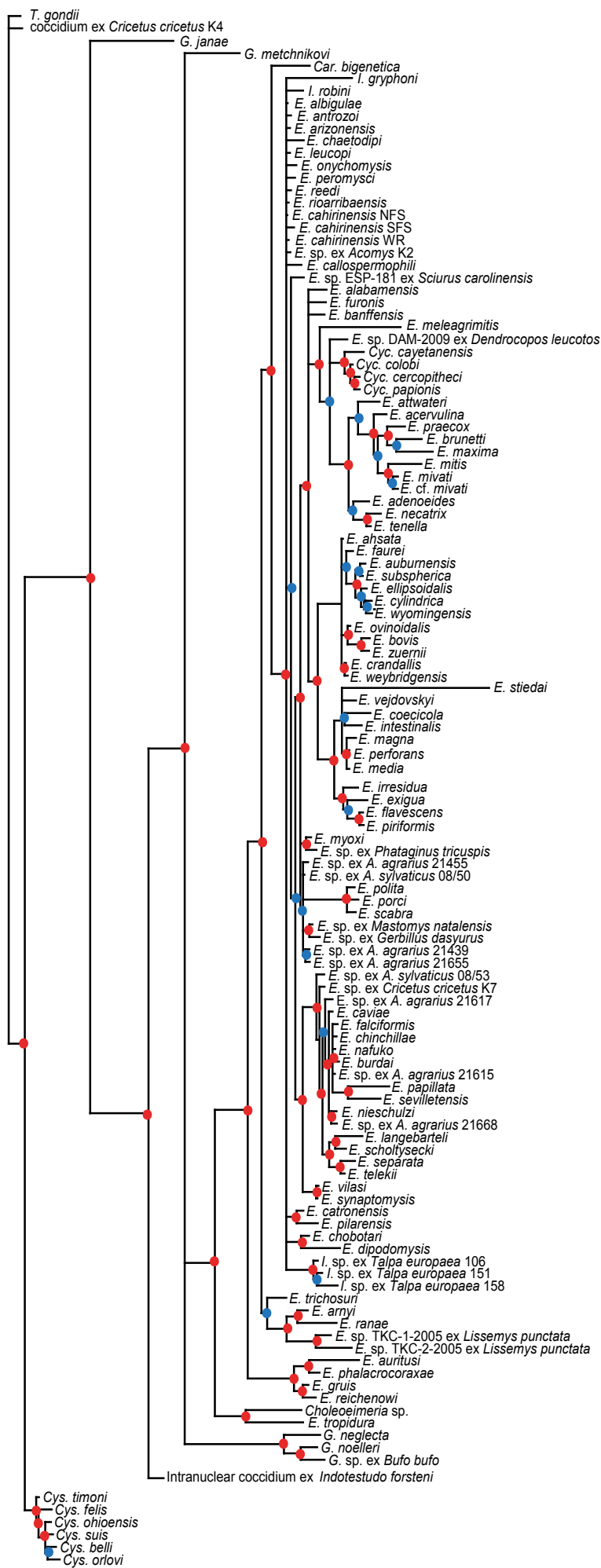

Supplement: Figure S4 — 18S rDNA BI tree. Strongly supported nodes (posterior probabilities >80%) are denoted by solid red circles. Nodes with posterior probabilities of 50–79% are marked with solid blue circles. (PDF) [file pone.0063601.s004.pdf]

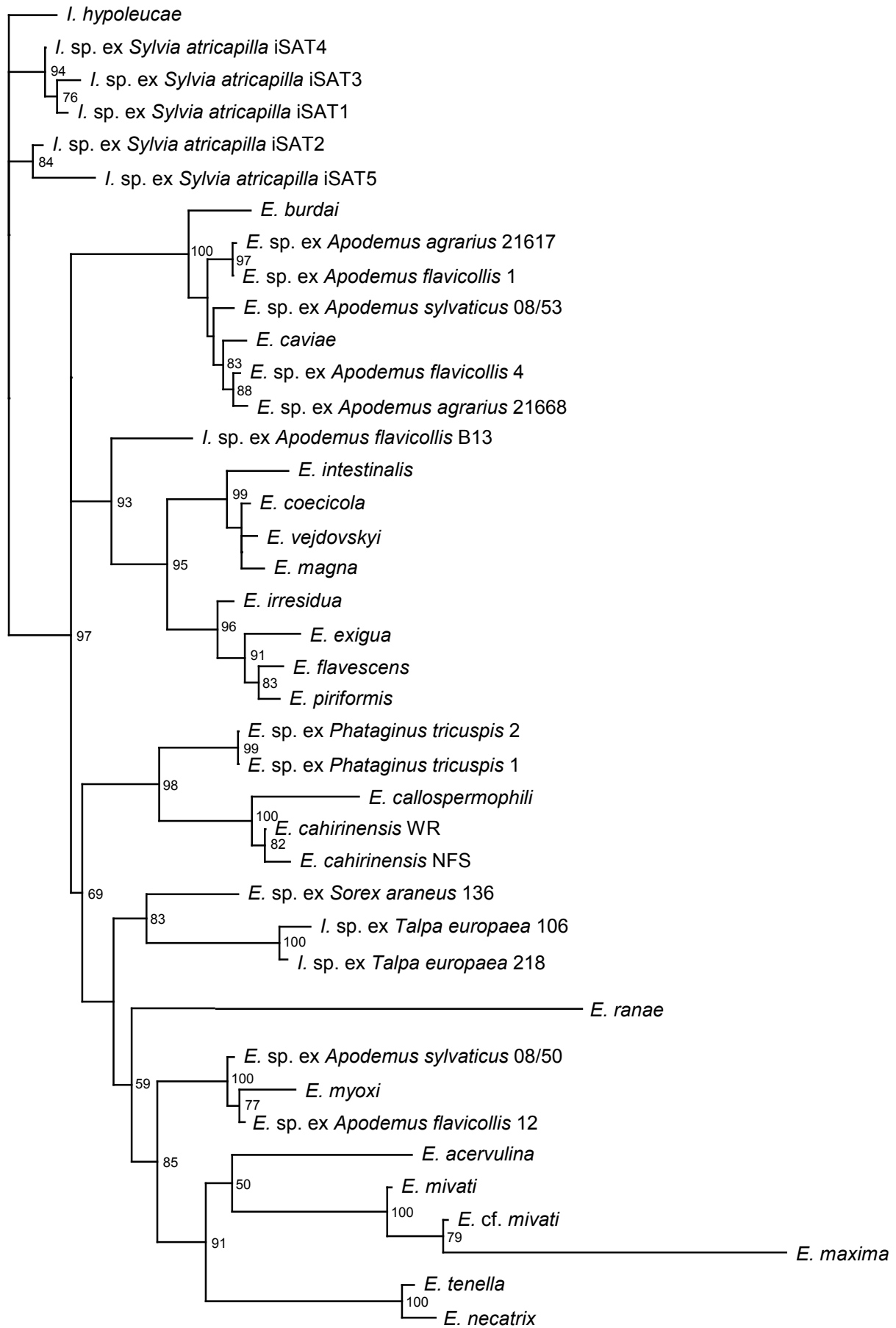

Supplement: Figure S5 — COI ML tree. Strongly supported nodes (bootstrap supports >80%) are denoted by solid red circles. Nodes with bootstrap supports of 50–79% are marked with solid blue circles. (PDF) [file pone.0063601.s005.pdf]

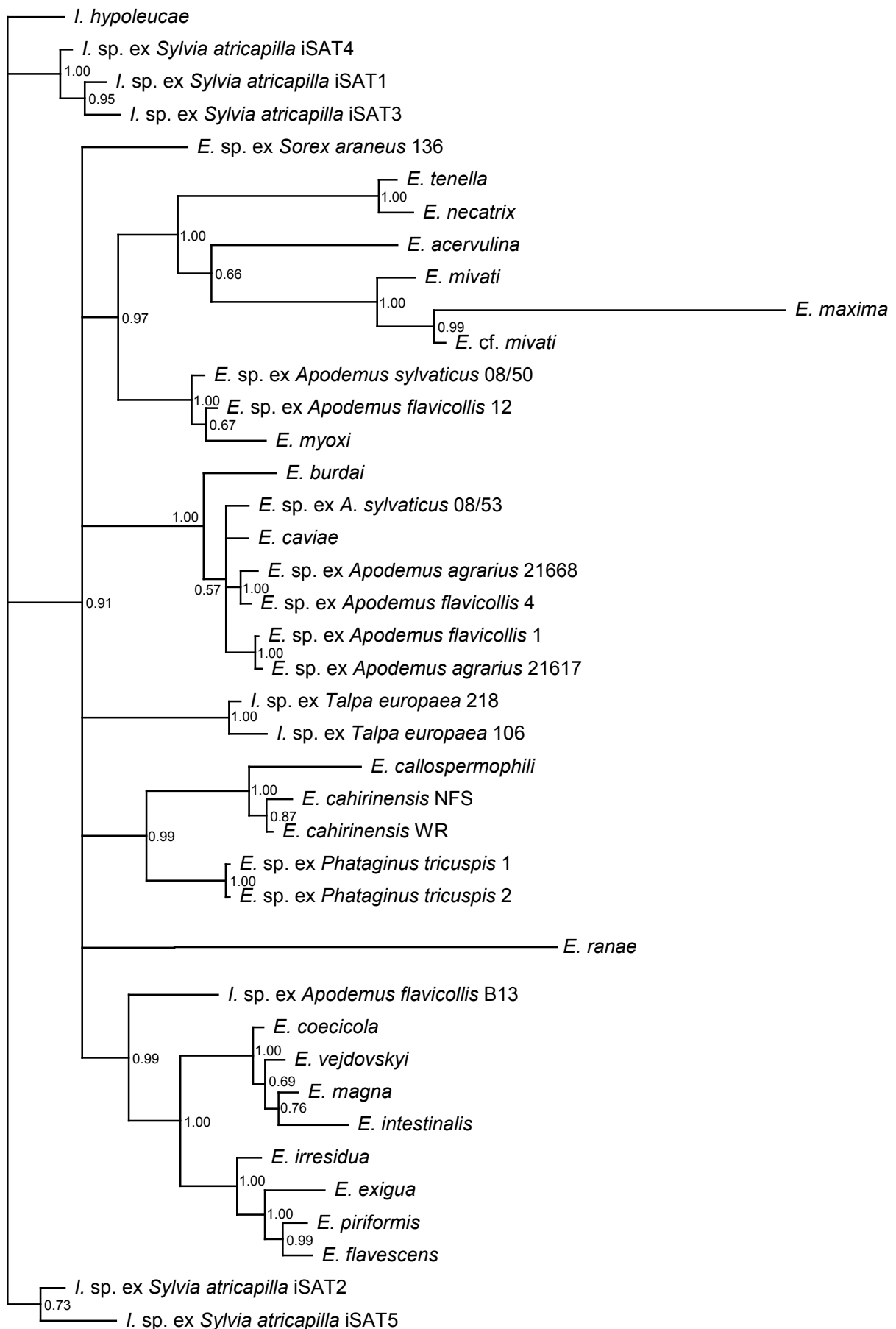

0.1

Supplement: Figure S6 — COI BI tree. Strongly supported nodes (posterior probabilities >80%) are denoted by solid red circles. Nodes with posterior probabilities of 50–79% are marked with solid blue circles. (PDF) [file pone.0063601.s006.pdf]

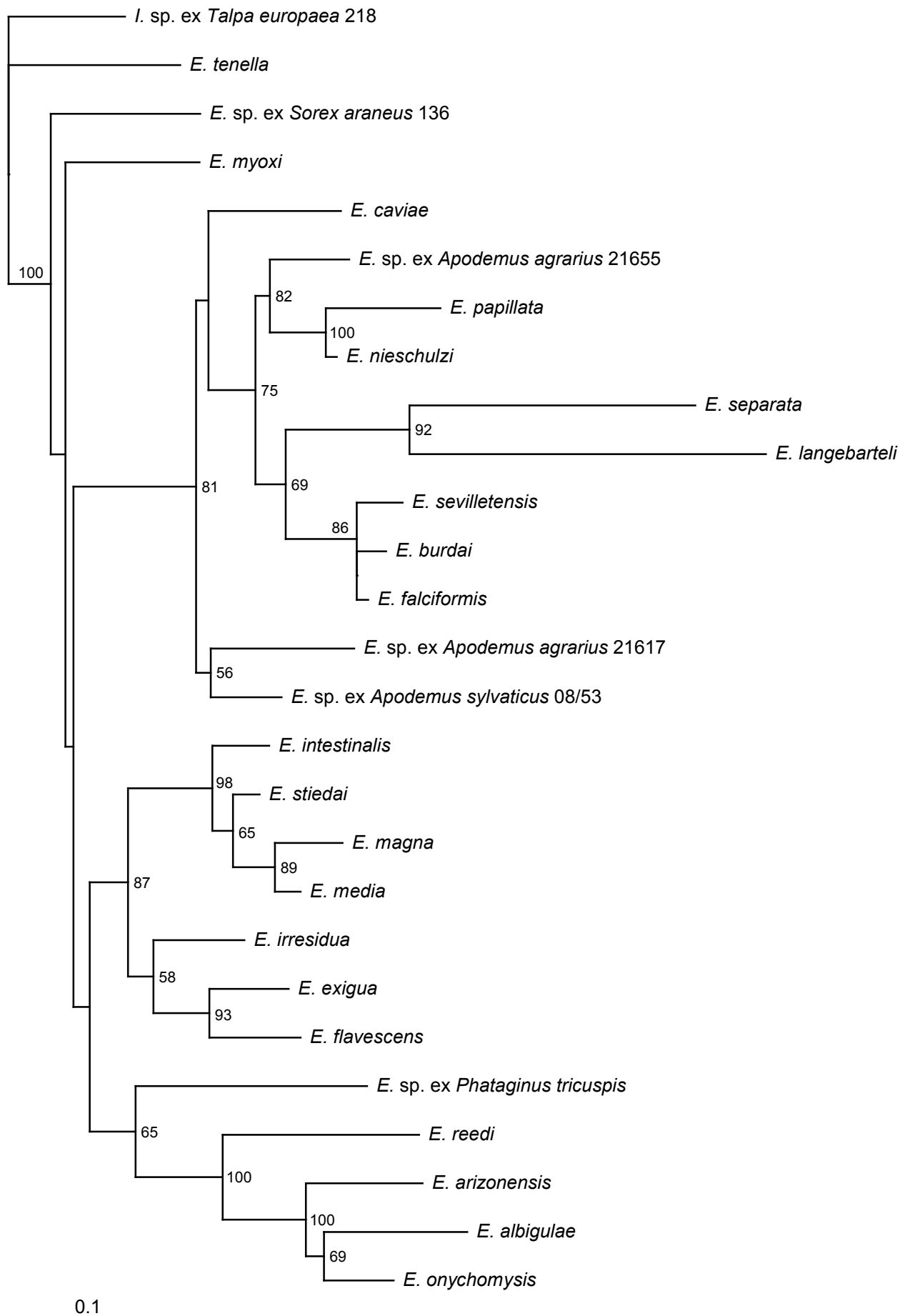

Supplement: Figure S7 — ORF 470 ML tree. Strongly supported nodes (bootstrap supports >80%) are denoted by solid red circles. Nodes with bootstrap supports of 50–79% are marked with solid blue circles. (PDF) [file pone.0063601.s007.pdf]

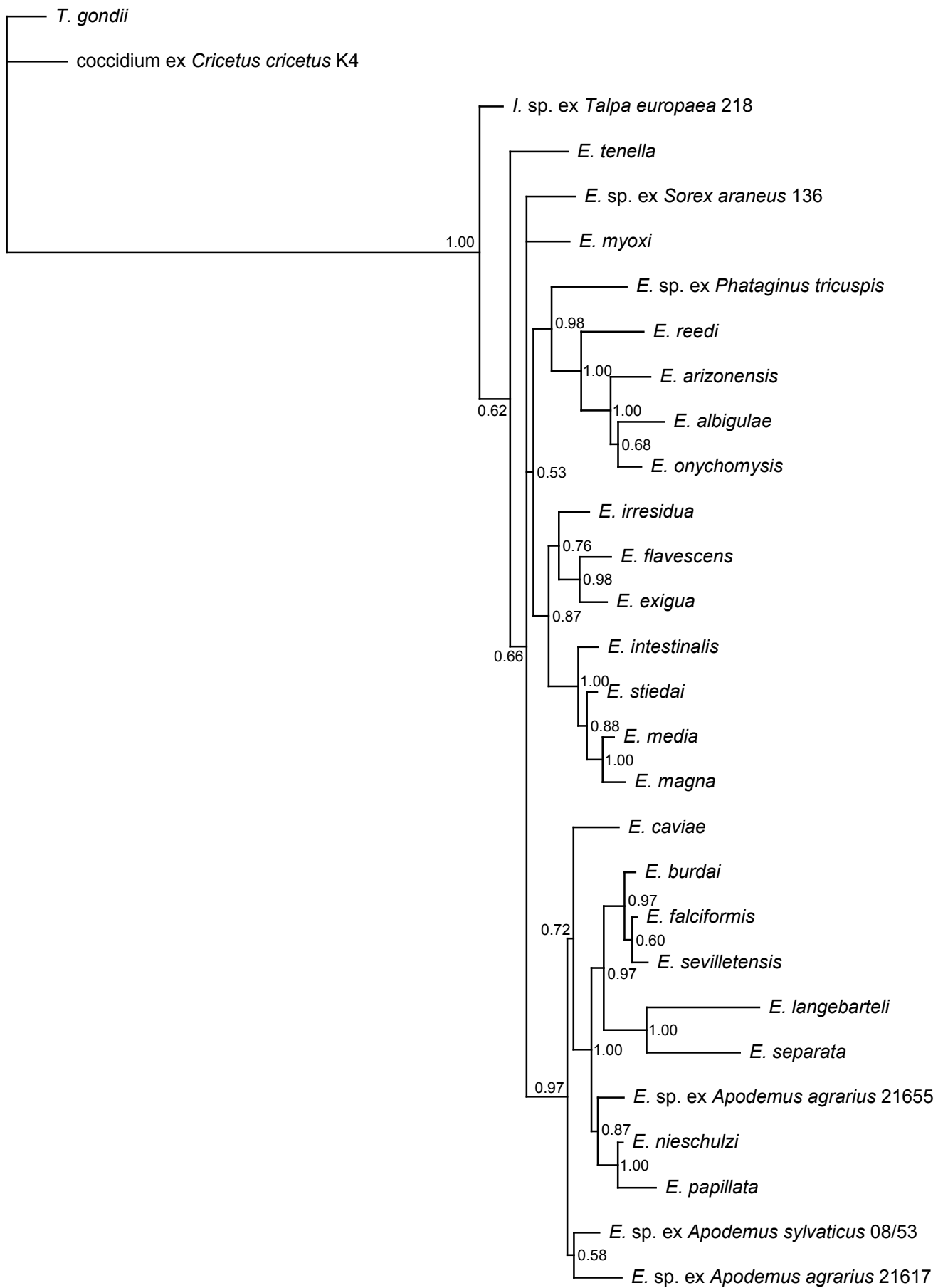

Supplement: Figure S8 — ORF 470 BI tree. Strongly supported nodes (posterior probabilities >80%) are denoted by solid red circles. Nodes with posterior probabilities of 50–79% are marked with solid blue circles. (PDF) [file pone.0063601.s008.pdf]
